# Bone status of children born from mothers with autoimmune diseases treated during pregnancy with prednisone and/or low molecular weight heparin

**DOI:** 10.1186/1546-0096-12-47

**Published:** 2014-10-23

**Authors:** Ilaria Pagnini, Gabriele Simonini, Loredana Cavalli, Giancarlo la Marca, Annamaria Iuliano, Maria Luisa Brandi, Francesca Bellisai, Bruno Frediani, Mauro Galeazzi, Luca Cantarini, Rolando Cimaz

**Affiliations:** Department of Pediatrics, University of Florence, AOU Meyer, Rheumatology Unit, Viale Pieraccini 24, 50139 Florence, Italy; Department of Endocrinology, University of Florence, Florence, Italy; Department of Rheumatology, Policlinico Le Scotte, University of Siena, Siena, Italy

**Keywords:** Osteoporosis, Autoimmune diseases, Heparin, Pregnancy, Corticosteroids

## Abstract

**Background:**

To evaluate bone status in children born from mothers followed for autoimmune diseases and treated during pregnancy with low molecular weight heparin (LMVH) and/or prednisone.

**Findings:**

History, physical examination, laboratory tests and phalangeal ultrasonography were performed. Demographic, clinical, and laboratory data were entered into a customized database, and results were analyzed with SPSS software. In children whose mothers were treated with LMWH, we retrieved dried blood spots taken for newborn screening, and analyzed the presence of heparin with tandem mass spectrometry.

We enrolled 27 females and 14 males born from 31 mothers with SLE or connective tissue diseases. These women were continuously treated during pregnancy with LMWH (n = 10), prednisone (n = 16), or both (n = 15). Bone ultrasound revealed low values (≤3 centile for age) in ten patients. In a multistep regression analysis, age at examination resulted the single predictor of low ultrasound values (p < 0.004). Tandem mass spectroscopy failed to determine traces of heparin in newborn blood.

**Conclusions:**

Children born from mothers with autoimmune diseases are at risk to develop reduced bone mass. The administration of LMWH and of prednisone seems to be safe with regard to children’s bone health.

## Background

Pregnancy is characterized by the combination of a procoagulant state, meant to avoid massive maternal bleeding during delivery, and venous stasis caused by venous dilation and compression by the gravid uterus [1]. Thus, pregnant women are at higher risk of developing thromboembolic complications; this risk is further increased among women suffering from prothrombotic conditions such as systemic autoimmune disorders [2]. Among patients affected with connective tissue diseases, thrombosis is a major issue in women with systemic lupus erythematosus (SLE), and in those with antiphospholipid syndrome (APL). Thus thromboprophylaxis with low-molecular-weight heparins (LMWH) is advised, although the potential fetal and maternal toxicity of antithrombotic drugs and their impact on delivery still needs to be fully elucidated. Concomitant treatment with low dose aspirin is generally also recommended [3]. Moreover, in patients with connective tissue diseases, corticosteroids such as prednisone and prednisolone are usually administered in order to control maternal disease flares.

Bone health is influenced by genetic and environmental factors, some of which concern early childhood or even prenatal life [4–6]. In this regard, chronic maternal disease and maternal treatments could potentially have an impact on bone status of their offspring.

Both long-term LMWH therapy and corticosteroids may be associated with osteopenia in mothers, although calcium and vitamin D supplementation seems to reduce the risk of drug-induced osteoporosis during pregnancy [7–11]. Although it has been shown that LMWHs do not seem to cross the placenta [12] and that they seem to be safe for the fetus, and although it is known that prednisone and prednisolone are mostly inactivated by placental hydroxilase, their effects on bone status in children born from mothers treated with these drugs during pregnancy are currently unknown.

The aim of our study was to evaluate bone status in a cohort of children born from mothers affected by systemic autoimmune disorders and treated during pregnancy with agents (prednisone and heparin) known to reduce bone mass.

## Methods

### Patients

Our study group included 41 children born from 31 mothers affected by systemic autoimmune diseases, who were treated with LMWH and/or prednisone throughout pregnancy. All mothers had been prospectively followed at the Rheumatology Unit of University of Siena, Italy from 2000 to 2011. Their medical records were reviewed and relevant demographic, clinical and laboratory data were collected into a customized database. Families were contacted and agreed to participate in the study. All children were evaluated at the Pediatric Rheumatology Unit of Meyer Children’s Hospital, Florence, Italy. A complete history and physical examination was performed in all cases. Body mass index (BMI) was calculated with the following formula: weight (kg)/height (m)^2^. Relevant data were also entered into the database. Informed consent by parents, and Institutional Ethics Committee approval were obtained (Policlinico “Le Scotte”, Siena, Italy).

### Laboratory assessment

In all children complete blood count, C-reactive protein, serum calcium, phosphorus and creatinine were determined at clinic visit. Bone formation markers (bone alkaline phosphatase, osteocalcin), a bone resorption marker [serum carboxyterminal telopeptide of type I collagen (ICTP)], serum vitamin D levels (25-OH Vit. D), as well as autoantibodies [antinuclear antibodies (ANA), and anticardiolipin antibodies (aCL)] were also determined. Bone metabolism markers and aCL were measured by ELISA, while ANA were determined by indirect immunofluorescence.

### Bone status assessment

Phalangeal ultrasonography (DBM Sonic 1200 machine, IGEA, Carpi, Italy) was performed in all patients. The machine was calibrated at the start of each session. The measurements were made by one trained operator (LC) in order to avoid interoperator errors. A phantom arm was used to calibrate measures and the procedures were always performed in standard room temperature and by the same operator (LC).

The technology is based on the transmission of ultrasound through the proximal phalanges, using a gel based device. The device calculates the amplitude-dependent speed of sound (AD-SoS) and averages the AD-SoS values for the four phalanges measured, and the intra-operator errors are less than 1% [13, 14]. The DBM Sonic device consists of two spring-loaded calipers, which are attached to probes. The probes were positioned medio-laterally on the proximal phalanx. An ultrasound pulse is transmitted from one probe and received by the other after passing through the phalanx. A mean amplitude-dependent speed of sound value is automatically generated on a screen that reflects the quality of the bone the ultrasound has passed through. The calipers are adjusted around the phalanx to give an optimal signal. Values below the given threshold for soft tissue measurement were excluded. The second to fifth fingers of each hand were measured to give a mean AD-SoS value for each hand. This was compared to the manufacturer’s database to derive a Z score that reflected the standard deviation from the age- and sex-matched mean value; [15] results were expressed as percentiles.

### Statistical analysis

All results are expressed as mean ± standard deviation (SD) or median (range). Quantitative ultrasound (QUS) percentile values (spanning between 3rd and 97th percentiles) were provided by the manufacturer, and each one was also adjusted for the BMI values at the time of bone ultrasound determination.

Mann–Whitney *U*-test, or Fisher’s exact test, were used as appropriate. Nonparametric tests were used, where necessary, due to the small size of our groups and to the skewness of data. Levels of p < 0.05 were considered statistically significant. Analyses were performed on SPSS package for Windows, version 13.0 (SPSS, Inc., Chicago, IL, USA).

The Spearman rank correlation test was used to determine correlation coefficients between QUS percentiles and the following variables: gender, age, weight, height, BMI, family history of autoimmune diseases, maternal diagnosis, maternal treatment during pregnancy (both as categorical variable, i.e. receiving treatment during pregnancy or not, as well as specific treatment received), being sibling or only child, intrauterine complications, APGAR score, cesarean section, length, weight and cranial circumference at birth, prematurity, formula or breast feeding, growth and development during the first year of life, vitamin D supplementation, vitamin D levels, osteocalcin and bone alkaline phosphatase serum levels.

Multiple stepwise regression, with BMI values at the time of QUS evaluation acting as covariate, was performed to determine variables that could correlate independently; thus the predictors used in the final model were those showing a significant correlation in the univariate analysis.

### Sample preparation for mass spectrometry analysis

In order to exclude transplacental passage of LMWH, we were able to retrieve dried blood spots (DBS), taken after 48 hours of life for newborn screening purposes, from children whose mothers received LMWH during pregnancy and we investigated the possible presence of heparin with a new liquid chromatography tandem mass spectrometry method.

Sample preparation was performed according to published and validated method for glycosaminoglycans measurement with slight modifications [16]. Heparin was measured as methylated disaccharides after methanolysis. Methanolysis was performed on extracted DBS (3.2 mm containing about 3.4 μL of blood) by using 200 μL HPLC water. For heparin, among all degradation products detected, we chose one particular disaccharide in order to obtain the highest specificity by liquid chromatography and mass spectrometry. The samples were measured using analytical HPLC coupled to a QTRAP 5500 (AB SCIEX, Toronto, Canada) equipped with the TurboIonSpray source operated in positive ion mode. The capillary voltage of the mass spectrometer was set to 5500 V, the “turbo” gas flow was 10 L/min of air heated at 550°C. The following transitions were monitored in MRM mode (Multiple Reaction Monitoring): *m*/*z* 384.2.1 > 162.1. Optimal CE (Collision Energy) and CXP (Collision Cell Exit Potential) were found at 20 Volts and 13 Volts respectively. The resulting DP (Declustering Potential) was +40 Volts. The quantitation experiments were undertaken with an external calibration by using a Series 1290 Infinity LC System (Agilent Technologies, Waldbronn, Germany) HPLC Capillary Pump coupled to an Agilent Micro ALS autosampler, both being fully controlled from the QTRAP 5500 data system. Liquid chromatography was performed using a Kinetek 2.6 μm C18 100 Å 7.5 × 3 mm4 HPLC column (Phenomenex, Andover, USA). Column flow was 0.2 mL/min using a water/acetonitrile (20:80) and 0.05% formic acid in an isocratic elution system. The eluent from the column was directed to the TurboIonSpray probe without split ratio. Three μL of the extracted sample were injected for the HPLC-MS/MS experiments. System control and data acquisition were performed with Analyst 1.5.1 software including the “Explore” option (for chromatographic and spectral interpretation) and the “Quantitate” option (for quantitative information generation). Calibration curves were constructed with the Analyst Quantitation program using a linear least-square non-weighted regression.

## Findings

We enrolled 27 girls and 14 boys (mean age at clinic visit 5 years and 10 months, range 9 months- 12 years), born from 31 mothers with systemic autoimmune diseases (there were 9 enrolled mothers who had two pregnancies during which prednisone and/or LMWH were administered).

All mothers had been continuously treated during all pregnancies with daily LMWH in 10 cases, prednisone in 15 cases, or both in 15 cases. There were 11 preterm deliveries (gestational age < 37 weeks), in 7 women affected by SLE, 3 by primary antiphospholipid syndrome (PAPS) and one by granulomatosis with polyangitis; fetal distress was reported in 4 cases. Median birth weight was 2935 g, range 520–3790. Eight newborns had neonatal complications: respiratory distress (n = 3), jaundice (n = 3), transient hypocalcemia and hypoxic-ischemic syndrome (n = 1 each). Breastfeeding for at least 6 months was reported in 12 cases; 37 children had received vitamin D supplementation (400 IU/day; 6/37 for 6 months, 31/37 for the first year of life), and growth and development were within normal limits. No history of fractures in mothers or children was recorded.

In all children clinical examination was within normal limits for age. Of note, 2 patients had alterations on primary teeth, with cavities and enamel abnormalities, but without any damage on permanent teeth; one of these babies was born from a mother with SLE treated with LMWH and prednisone and the other one, with neonatal transient hypocalcemia, was born from a woman with granulomatosis and polyangitis who had received prednisone.

Table 1 shows main clinical, epidemiological, laboratory, and instrumental results collected from both mothers and their children.

**Table 1:** **Main clinical, epidemiological, laboratory and instrumental results collected from mothers with autoimmune diseases and their children**

Mother	Pregnancy	Diagnosis in mothers	Autoantibody positivity in mothers	Treatment during pregnancy	Concomitant medications	Gestational age (weeks + days)	Post-partum complications	Child age at clinic visit (years and months)	BMI at visit	QUS (percentile)
1	a	PAPS	none	Enoxiparin 2000 UI bid.	Aspirin	38	None	7 yrs 6 mo	14.9	97
1	b	PAPS	none	Enoxiparin 2000 UI bid.	Aspirin	36	None	9 yrs	17.3	75
2	a	Sjögren	ANA	Enoxiparin 2000 UI bid.	None	40	None	8 yrs 9 mo	15.9	75
3	a	SLE	ANA, ENA	Enoxiparin 2000 UI bid.	Aspirin	30 + 4	None	5 yrs 1 mo	16.9	75-90
3	b	SLE	ANA, ENA	Enoxiparin 2000 UI bid.	Aspirin	36 + 5	None	2 yrs	16.1	< 3
4	a	UCTD	ANA, LAC	Enoxiparin 2000 UI bid.	Aspirin	39 + 4	None	6 yrs 2 mo	16.8	97
5	a	PAPS	ANA, ENA, LAC, aCL IgM	Nadroparin 3800 UI daily	Aspirin	39 + 4	None	5 yrs 3 mo	15.4	25
6	a	Systemic Sclerosis	ANA, aCL IgM	Nadroparin3800 UI daily	None	38 + 4	None	6 yrs 4 mo	16.1	< 3
6	b	Systemic Sclerosis	ANA, aCL IgM	Nadroparin3800 UI daily	None	38	None	9 yrs 4 mo	19.5	< 25
7	a	SLE	ANA, ENA, LAC, aCL IgM, aCL IgG	Enoxiparin 4000 UI daily	Aspirin	40	None	3 yrs 9 mo	17.2	< 3
8	a	SLE	ANA, aCL IgM	Methylprednisolone 4 mg daily	None	40	None	2 yrs 3 mo	17.1	> 97
9	a	MCTD	ANA, ENA	Methylprednisolone 4 mg daily	None	40	None	6 yrs 9 mo	15.8	75
10	a	Granulomatosis with polyangitis	ANA, ANCA	Prednisone 15 mg daily	IVIG	40	None	7 yrs 9 mo	16.5	25
10	b	Granulomatosis with polyangitis	ANA, ANCA	Prednisone 15 mg daily	IVIG	40	None	9 yrs	24	75
11	a	SLE	ANA, anti-DNA, aCL IgG	Prednisone 25 mg daily	IVIG	36	Post-partum bleeding	8 yrs 3 mo	14.4	50
12	b	SLE	ANA, ENA, anti-DNA, aCL IgG, aCL IgM	Prednisone 25 mg daily	Aspirin	37 + 1	None	1 yrs 4 mo	16.4	25
13	a	SLE	ANA	Prednisone 25 mg daily	None	36 + 4	None	9 yrs 3 mo	16.7	50
13	b	SLE	ANA	Prednisone 25 mg daily	None	39	None	10 yrs 7 mo	18.9	75
14	a	SLE	ANA	Prednisone 5 mg daily	Aspirin	40	None	5 yrs 5 mo	15.4	50
15	b	Sjögren	ANA, ENA	Prednisone 5 mg daily	None	40	None	2 yrs 4 mo	12.4	50
16	a	RA	None	Prednisone 5 mg daily	None	39	None	12 yrs	14.5	25
16	b	RA	None	Prednisone 5 mg daily	None	40	None	4 yrs 5 mo	14	75
17	a	SLE	ANA, ENA, anti-DNA	Prednisone 5 mg daily	Aspirin	40	None	6 yrs 7 mo	12.2	25
17	b	SLE	ANA, ENA, anti-DNA	Prednisone 5 mg daily	Aspirin	40	None	3 yrs 9 mo	14	50
18	a	UCTD	ANA	Methylprednisolone 5 mg daily	None	39 + 5	None	1 yrs	16.2	< 3
19	b	SLE	ANA	Methylprednisolone 4 mg daily	Aspirin	40	None	6 yrs 4 mo	21.2	50
20	a	SLE	ANA, anti-DNA	Enoxiparin 4000 UI daily + Prednisolone 4 mg daily	None	40	None	10 yrs 9 mo	25.7	75
21	a	PAPS	ACL IgG, anti-β2GPI	Nadroparin 3800 UI daily + Prednisone 5 mg daily	None	34	Mitralic insufficiency	5 yrs 10 mo	15	25
21	b	PAPS	ACL IgG, anti-β2GPI	Nadroparin3800 UI daily + Prednisone 5 mg daily	None	34	Thrombocytopenia	9 yrs 7 mo	20.1	25-50
22	a	SLE	ANA, ENA, anti-DNA	Nadroparin2850 UI daily + Methylprednisolone 4 mg daily	None	38	None	11 yrs 3 mo	17.5	97
22	b	SLE	ANA, ENA, anti-DNA	Nadroparin2850 UI daily + Methylprednisolone 4 mg daily	None	36 + 5	None	5 yrs 5 mo	15.8	< 3
23	a	MCTD	ENA, aCL IgG	Enoxiparin 4000 UI daily + Prednisone 5 mg daily	None	37 + 1	None	9 mo	17.5	25
24	a	SLE	ANA, anti-DNA, aCL IgG, anti-β2GPI	Enoxiparin 4000 UI daily + Prednisone 10 mg daily	IVIG + Aspirin	40	None	10 yrs	25	< 3
24	b	SLE	ANA, anti-DNA, aCL IgG, anti-β2GPI	Enoxiparin 4000 UI daily + Prednisone 10 mg daily	IVIG + Aspirin	38	None	4 yrs 7 mo	15.4	< 3
25	a	SLE	ANA	Enoxiparin 4000 UI daily + Prednisone 25 mg daily	IVIG	40	None	1 yrs 5 mo	15.3	< 3
26	a	UCTD	ENA, aCL IgM	Enoxiparin 4000 UI daily + Prednisone 5 mg daily	None	36	None	10 yrs 9 mo	16.6	25
27	a	UCTD	ENA	Enoxiparin 4000 UI daily + Prednisone 5 mg daily	Aspirin	40	None	9 mo	16	97
28	a	UCTD	ENA, anti-β2GPI	Enoxiparin 4000 UI daily + Prednisone 5 mg daily	Aspirin	37	None	2 yrs 3 mo	19.1	97
29	a	SLE	ANA, ENA, LAC, anti-DNA	Enoxiparin 4000 UI daily + Prednisone 5 mg daily	None	40	None	5 yrs 11 mo	21.8	25
30	a	SLE	ANA	Nadroparin2850 UI daily + Prednisolone 8 mg daily	None	34	Hepatogestosis	11 mo	15.6	25
31	a	SLE	ANA	Nadroparin2850 UI daily + Prednisolone 8 mg daily	None	25 + 1	Hepatogestosis	5 yrs	13.8	25

Routine laboratory tests were in the normal range in all cases. Despite previous routine supplementation, vitamin D serum levels were in the normal range (>30 ng/ml) in only 15/41 patients, while they were decreased in 26 children; these had been exposed to LMWH (n = 6), prednisone (n = 12), and LMWH and prednisone (n = 8). Bone formation and resorption markers were in all cases in the normal range provided by our laboratory. Autoantibodies (ANA, aCL IgM and IgG), at the moment of evaluation, were present in 13 cases (9 children were ANA-positive, and 4 aCL-positive), all born from mothers with the same autoantibodies.

Bone ultrasound recorded QUS values ≤3 percentile for age and gender in 10 children, 10 resulted between >3° and ≤25° and all the other 21 showed a QUS percentile value higher than 25°.

**Figure 1:**
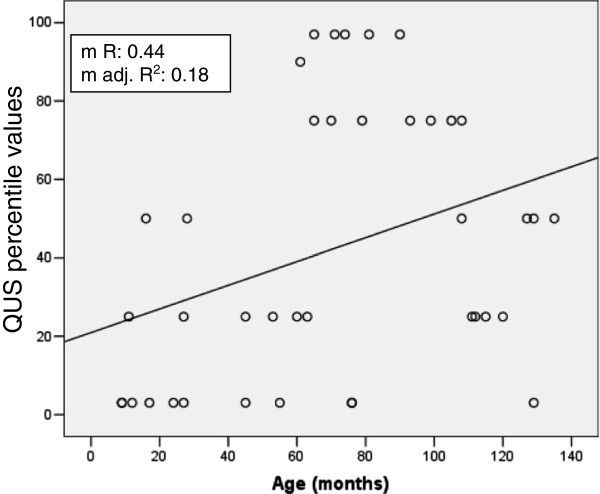
**The correlation of quantitative ultrasound (QUS) percentile values with age, in months, at the time of the bone ultrasound examination is shown in a multi-stepwise regression analysis model (p < 0.03).**

**Figure 2:**
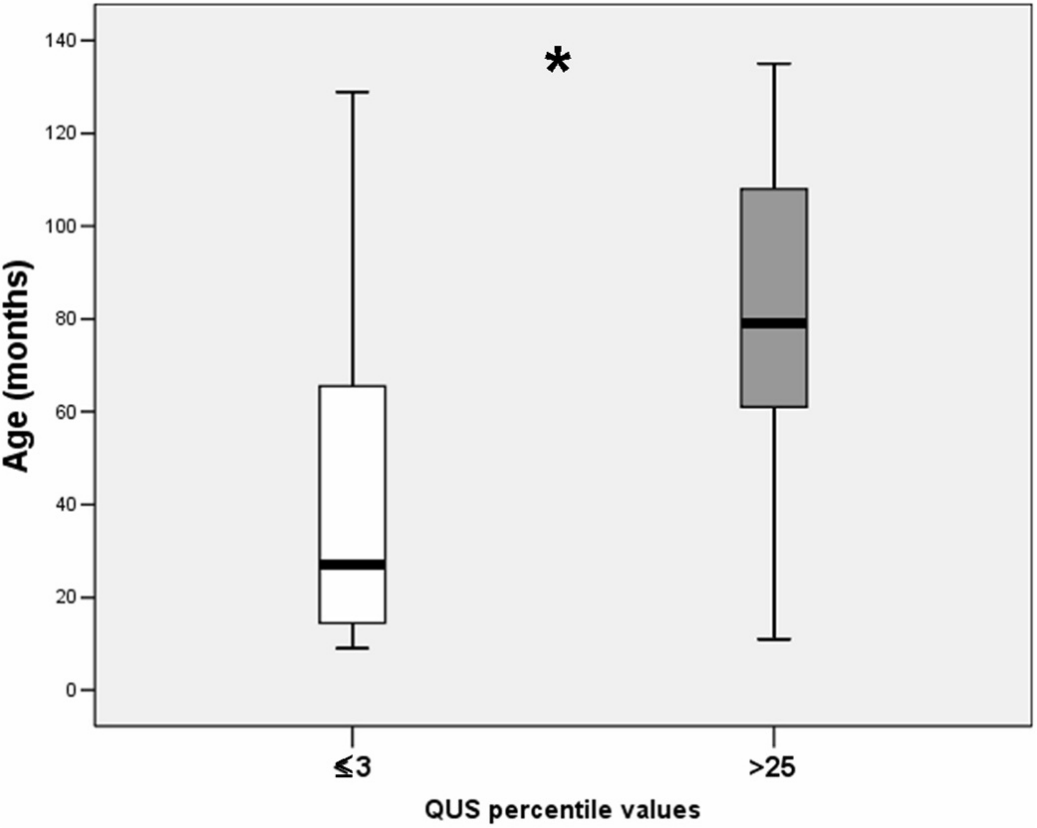
**Age (in months) at the time of bone ultrasound examination in children with ≤3 percentile QUS values (white box) and in children with >25° percentile QUS values (grey-box).** The central line represents the distribution median, boxes span 25th to 75th percentiles, and error bars extend from 10th to 90th percentiles. * = p <0.006.

In univariate analysis, QUS percentile values showed a significant correlation with age at examination (r_s_: 0.41, p < 0.006), and osteocalcin values (r_s_: -0.32, p < 0.04). No significant correlations were detected with the other entered variables, including heparin and steroid use during pregnancy. In multi-stepwise regression analysis, weighted for BMI values at the time of bone ultrasound determination, age at examination resulted the single predictor of QUS values (multiple R = 0.44, multiple adjusted R^2^: 0.18, p < 0.03) (Figure 1). In children with low (≤3 percentile) QUS values, age at examination resulted lower than in children with normal (>25 percentile) QUS values (median, range: 27 months, 9–129 *vs* 79 months, 11–135, p < 0.006) (Figure 2).

Tandem mass spectroscopy performed on DBS failed to determine traces of heparin in newborn blood.

## Discussion

Many factors influence the accumulation of bone mineral during childhood and adolescence, including heredity, gender, diet, physical activity and endocrine status [17, 18]. Measures for maximizing bone mineral acquisition, particularly through physical activity and adequate dietary calcium intake, are likely to affect the risk of fracture in later life. In addition to these modifiable factors during childhood, evidence has also shown that the risk of fracture might be programmed during intrauterine life. Maternal smoking, diet (particularly vitamin D deficiency), physical activity, diseases and drugs also appear to modulate bone mineral acquisition in intrauterine life; furthermore, birthweight, weight in infancy, poor childhood growth seem also to be linked to adult bone mass [4–6, 19, 20].

Systemic autoimmune disorders largely affect women during their reproductive age. Pregnancy in connective tissue diseases has long been associated with poor obstetric outcomes, and can potentially affect disease activity as well. For example, in SLE the risk of spontaneous abortion, intrauterine fetal death, preeclampsia, intrauterine growth restriction and preterm birth is common, such as an active lupus nephritis in mothers [21]. So, a close follow-up is mandatory, including repeated laboratory test and serial ultrasonography in mothers, fetal surveillance tests, and fetal echocardiography for mothers with SS-A (Ro) or SS-B (La) antibodies [22]. In women with primary antiphospholipid syndrome (PAPS) the risk of pregnancy complications increases, including spontaneous miscarriages that typically occur after 10 weeks of gestation, midpregnancy fetal distress, intrauterine growth restriction, prematurity and preeclampsia. In others autoimmune conditions, such as rheumatoid arthritis (RA), systemic sclerosis and Sjögren syndrome the risk of adverse pregnancy outcome is variable.

Moreover, women affected by autoimmune diseases are at high risk of developing osteopenia, osteoporosis and fractures through both disease-specific (chronic arthritis, reduced physical activity, induction of cytokines promoting bone resorption, renal impairment, endocrine factors) and nondisease-specific mechanisms (chronic anticoagulant, glucocorticoid and immunosuppressive treatments, vitamin D deficiency). Regarding thromboprophylaxis, subcutaneous heparin is crucial against the risk of recurrent thromboembolism or pregnancy loss [23]. Heparin and its low molecular weight derivatives can be associated with bone loss and an increased risk of fractures; however, LMWHs seem to be less deleterious to bone than unfractionated heparin [24, 25]. In addition, pregnancy by itself and lactation may also predispose to osteopenia [26, 27].

Unfractionated heparin is a large molecule, so it does not cross the placenta. It seems not to be teratogenic and not cause toxic fetal effects. Several studies have confirmed the safety and efficacy of LMWH during pregnancy as well [28–34]. LMWHs also does not seem to cross the placenta [30, 31, 33], thus with a fetal safety profile equivalent to that of unfractionated heparin. However, the possibility that unfragmented heparin or LMWH pass the placental barrier to the fetus has been studied only indirectly. Mätzsch et al. studied 21 women who had an abortion by hysterotomy. Laboratory assessments included assays of factor Xa inhibitory activity (XaI), antithrombin III (ATIII) and a measurement of heparin-like substances in plasma with a competitive binding assay. The authors did not demonstrate any evidence for the passage of heparin or LMWH across the placental barrier, furthermore no differences were detected whether unfragmented heparin or LMWH had been given to the mothers [35]. In another study [12], 14 women who were going to have an early termination of pregnancy due to major fetal malformations were given 40 mg of enoxaparin. Maternal blood samples were drawn before and after the injection of enoxaparin, while fetal blood samples were taken only after the drug administration. Anti-IIa and anti-Xa activities were measured. A statistically significant increase of anti-Xa activity in the mothers studied was shown, while there was no detection of anti-IIa and anti-Xa activities in fetus. It was therefore concluded that enoxaparin does not cross the placenta and appeared to be safe for the fetuses. However, such studies and others [8, 35] measured only heparin activity in fetal plasma, and not direct drug presence. We were able to retrieve dried blood spots taken at birth from children whose mothers were administered with LMWH during pregnancy, and could investigate the possible presence of heparin with a new liquid chromatography tandem mass spectrometry method. Tandem mass spectroscopy performed on DBS failed to determine traces of heparin in newborn blood, thus confirming its fetal safety. With regard to corticosteroids, although a fetal effect of high-dose prednisone cannot be excluded, it is known that the fetus is protected from the effect of prednisone because of the placental enzyme 11-β-dehydrogenase, which oxidizes it into an inactive form.

In order to assess bone status, we performed phalangeal QUS, since it is a noninvasive method that provides a surrogate estimation not only of bone density, but also of bone elasticity and architecture, with specific parameters derived from sound waves passing through both cortical and trabecular bone [36–38]. The measurement site chosen was the hand phalanges, which are composed of predominantly cortical bone and are easily accessibile [39]. Although it is known that dual X-ray absorptiometry (DXA) is gold standard in order to asses bone density, its use in the pediatric age is hampered by the need to sedate young children and by radiation dose, albeit minimal. Moreover, we and others have shown a good correlation between ultrasound and DXA in pediatric rheumatic diseases [40–43].

Despite the fact that phalangeal QUS measurements have shown the ability to reveal changes due to skeletal growth [44], it is known that bone density values can artificially be modified by bone or body size. Hence, we have transformed QUS results by correcting centile values by BMI, which takes into account both height and weight. In our statistical analysis we have considered all factors that could be confounders for reduced bone density, such as birth weight [20], gender [18], intrauterine complications [5], prematurity, vitamin D supplementation and levels. In the multivariate analysis only age at examination resulted the single predictor of QUS values, in that older children had mostly normal values while younger infants did not. In particular, not only drugs did not seem to pass the placenta but also they were not linked to a low bone mass.

We hypothesize that mothers with autoimmune systemic diseases are at risk for delivering babies with low bone mass, by multiple mechanisms; however, the prognosis for these children with regard to their bone health seems to be good, since when tested during late childhood or preadolescence bone ultrasound values were in the normal range. We acknowledge the limit of the lack of a control group; however, QUS values for healthy children have already been obtained by our laboratory and did correlate with the reference values from the manufacturer that we have used. In addition, we did not have bone marker values from age-matched healthy controls, but most of the results in our series were already in the normal range provided by our laboratory, hence ruling out the possibility of false positives due to high bone turnover in a growing skeleton.

## Conclusions

In conclusion, our study suggests that children born from mothers with autoimmune diseases are at risk for low bone mass during the first years of life, and shows that this risk is not linked to exposure to potentially osteotoxic drugs. Moreover, we have confirmed for the first time with a direct assay that LMWHs do not cross the placental barrier.

## Abbreviations

PAPS: Primary antiphospholipid syndrome
SLE: Systemic lupus erythematosus
UCTD: Undifferentiated connective tissue disease
MCTD: Mixed connective tissue disease
RA: Rheumathoid arthritis
IVIG: intravenous immunoglobulins
ANA: Antinuclear antibodies
ENA: Extractable nuclear antigens
ANCA: Antineutrophil cytoplasmic antibodies
LAC: Lupus anticoagulant
aCL: Anticardiolipin antibodies
aPL: Antiphospholipid antibodies.

## References

[1] Bazaan M, Donvito V (2001). Low-molecular-weight heparin during pregnancy. Throm Res.

[2] Ruitz-Irastorza G, Khamashta Munther A (2005). Management of thrombosis in antiphospholipid syndrome and systemic lupus erythematosus in pregnancy. Ann N Y Acad Sci.

[3] Tuthill JI, Khamashta MA (2009). Management of antiphospholipid syndrome. J Autoimmun.

[4] Cooper C, Westlake S, Harvey N, Javaid K, Dennison E, Hanson M (2006). Review: developmental origins of osteoporotic fracture. Osteoporosis Int.

[5] Gale CR, Martyn CN, Kellingray S, Eastell R, Cooper C (2001). Intrauterine programming of adult body composition. J Clin Endocrinol Metab.

[6] McGuigan FE, Murray L, Gallagher A, Davey-Smith G, Neville CE, Van't Hof R, Boreham C, Ralston SH (2002). Genetic and environmental determinants of peak bone mass in young men and women. J Bone Mineral Res.

[7] Rai R, Cohen H, Dave M, Regan L (1997). Randomised controlled trial of aspirin and aspirin plus heparin in pregnant women with recurrent miscarriage associated with phospholipid antibodies. BMJ.

[8] Deruelle P, Coulon C (2007). The use of low-molecular-weight heparins in pregnancy- how safe are they?. Curr Opin Obstet Gynecol.

[9] Rowan JA, McLintock C, Taylor RS, North RA (2003). Prophylactic and therapeutic enoxaparin during pregnancy: indications, outcomes and monitoring. Aust N Z J Obstet Gynaecol.

[10] Huxtable LM, Tafreshi MJ, Ondreyco SM (2005). A protocol for the use of enoxaparin during pregnancy: results from 85 pregnancies including 13 multiple gestation pregnancies. Clin Appl Thromb Hemost.

[11] Mok GC, Wong RWS (2001). Pregnancy in systemic lupus erythematosus. Postgrad Med J.

[12] Dimitrakakis C, Papageorgiou P, Papageorgiou I, Antzaklis A, Sakarelou N, Michalas S (2000). Absence of transplacental passage of the low molecular weight heparin enoxaparin. Haemostasis.

[13] Wuster C, Albanese C, DeAloysio D, Duboeuf F, Gambacciani M, Gonnelli S, Glüer CC, Hans D, Joly J, Reginster JY, De Terlizzi F, Cadossi R (2000). Phalangeal osteosonogrammetry study: age-related changes, diagnostic sensitivity, and discrimination power. The Phalangeal Osteosonogrammetry Study Group. J Bone Miner Res.

[14] Benitez CL, Schneider DL, Barret-Connor E, Sartoris DJ (2000). Hand ultrasound for osteoporosis screening in postmenopausal women. Osteoporos Int.

[15] Cadossi R, Cane V (1996). Pathways of transmission of ultrasound energy through the distal metaphysics of the second phalanx of pigs: an in vitro study. Osteoporosis Int.

[16] Auray-Blais C, Bhérer P, Gagnon R, Young SP, Zhang HH, An Y, Clarke JT, Millington DS (2011). Efficient analysis of urinary glycosaminoglycans by LC-MS/MS in mucopolysaccharidoses type I, II and VI. Mol Genet Metab.

[17] Javad MK, Cooper C (2002). Prenatal and childhood influences on osteoporosis. Best Pract Res Clin Endocrinol Metab.

[18] Eriksson JG, Kajante E, Osmond C, Thornburg K, Barker DJ (2010). Boys life dangerously in the womb. Am J Hum Biol.

[19] Cooper C, Harvey N, Javaid K, Hanson M, Dennison E (2008). Nestle Nutr Workshop Ser Pediatric Program.

[20] Baird J, Kurshid MA, Kim M, Harvey N, Dennison E, Cooper C (2011). Does birthweight predict bone mass in adulthood? A systemic review and meta-analysis. Osteoporos Int.

[21] Lin P, Bonaminio P, Ramsey-Goldamn R, Imboden J, Hellmann D, Stone J (2007). Pregnancy And Rheumatic Disease. Current Rheumatology Diagnosis And Treatment.

[22] Bear AN, Witter FR, Petri M (2011). Lupus and pregnancy. Obstet Gynecol Surv.

[23] Kher A, Bausersache R, Nielsen JD (2007). The management of thrombosis in pregnancy: role of low-molecular weight heparin. Thromb Haemost.

[24] Rajgopal R, Bear M, Butcher MK, Shaughnessy SG (2008). The effects of heparin and low molecular weight heparins on bone. Thromb Res.

[25] Rodger MA, Kahn SR, Cranney A, Hodsman A, Kovacs MJ, Clement AM, Lazo-Langner A, Hague WM, TIPPS investigators (2007). Long-term dalteparin in pregnancy not associated with a decrease in bone mineral density: substudy of a randomized controlled trial. J Thromb Haemost.

[26] Michalakis K, Peitsidis P, Ilias I (2011). Pregnancy- and lactation-associated osteoporosis: a narrative mini-review. Endocr Regul.

[27] Choe EY, Song JE, Park KH, Seok H, Lee EJ, Lim SK, Rhee Y (2011). Effect of teriparatide on pregnancy and lactation-associated osteoporosis with multiple vertebral fractures. J Bone Miner Metab.

[28] Sanson BJ, Lensing AW, Prins MH, Ginsberg JS, Barkagan ZS, Lavenne-Pardonge E, Brenner B, Dulitzky M, Nielsen JD, Boda Z, Turi S, Mac Gillavry MR, Hamulyák K, Theunissen IM, Hunt BJ, Büller HR (1999). Safety of low-molecular weight heparin in pregnancy: a systematic review. Thromb Haemost.

[29] Greer IA, Nelson-Piercy C (2005). Low-molecular-weight heparins for thromboprophylaxis and treatment of venous thromboembolism in pregnancy: a systematic review of safety and efficacy. Blood.

[30] Melissari E, Parker CJ, Wilson NV, Monte G, Kanthou C, Pemberton KD, Nicolaides KH, Barrett JJ, Kakkar VV (1992). Use of low molecular weight heparin in pregnancy. Thromb Haemost.

[31] Forestier F, Daffos E, Capell-pavlovsky M (1984). Low molecular weight heparin (PK 10169) does not cross the placenta during the second trimester of pregnancy. Study by direct fetal blood sampling under ultrasound. Thromb Res.

[32] Omri A, Delaloye JF, Bachmann F (1989). Low molecular weight heparin novo (LHN-1) does not cross the placenta during the second trimester of pregnancy. Thromb Haemostas.

[33] Forestier F, Daffos F, Rainauut M, Toulemonde F (1987). Low molecular weight heparin (CY216) does not cross the placenta during the third trimester of pregnancy. Thromb Haemostas.

[34] Andrew M, Boneu B, Cade J, Cerskus AL, Hirsh J, Jefferies A, Towell ME, Buchanan MR (1985). Placental transport of low molecular weight heparin in the pregnant sheep. Br J Haematol.

[35] Mätzsch T, Bergqvist D, Bergqvist A, Hodson S, Dawes J, Hedner U, Ostergaard P (1991). No transplacental passage of standard heparin or an enzymatically depolymerized low molecular weight heparin. Blood Coagul Fibrinolysis.

[36] Kaufman JJ, Einhorn TA (1993). Perspective: ultrasound assessment of bone. J Bone Miner Res.

[37] Genant HK, Engleke K, Fuerst T, Glüer CC, Grampp S, Harris ST, Jergas M, Lang T, Lu Y, Majumdar S, Mathur A, Takada M (1996). Noninvasive assessment of bone mineral and structure: state of the art. J Bone Miner Res.

[38] Glüer CC (1997). Quantitative ultrasound techniques for the assessment of osteoporosis: expert agreement on current status. J Bone Miner Res.

[39] Mann TS, McGregor AH, Patel R (2008). The correlation between phalangeal quantitative ultrasonography and dual energy X-ray absorptiometry in women with premature ovarian failure. Mcgill J Med.

[40] Falcini F, Bindi G, Ermini M, Galluzzi F, Poggi G, Rossi S, Masi L, Cimaz R, Brandi ML (2000). Comparison of quantitative calcaneal ultrasound and dual energy X-ray absorptiometry in the evaluation of osteoporotic risk in children with chronic rheumatic diseases. Calcif Tissue Int.

[41] Simonini G, Cimaz R, Falcini F (2005). Usefulness of bone ultrasound techniques in pediatric rheumatic diseases. J Reumatol.

[42] Hartman C, Shamir R, Eshach-Adiv O, Iosilevsky G, Brik R (2004). Assessment of osteoporosis by quantitative ultrasound versus dual energy X-ray absorptiometry in children with chronic rheumatic diseases. J Rheumatol.

[43] Njeh CF, Shaw N, Gardner-Medwin JM, Boivin CM, Southwood TR (2001). Use of quantitative ultrasound to assess bone status in children with juvenile idiopathic arthritis: a pilot study. J Clin Densitom.

[44] Halaba Z, Pluskiewicz W (1997). The assessment of development of bone mass in children by quantitative ultrasound through the proximal phalanxes of the hand. Ultrasound Med Biol.

